# Solid-Phase Synthesis of Amine/Carboxyl Substituted Prolines and Proline Homologues: Scope and Limitations

**DOI:** 10.3390/molecules21030350

**Published:** 2016-03-15

**Authors:** Ziniu Zhou, William L. Scott, Martin J. O’Donnell

**Affiliations:** 1 Department of Pharmaceutical Engineering, Zhejiang Pharmaceutical College, 888 Yinxian Avenue Eastern Section, Ningbo 315100, China; 2 Department of Chemistry and Chemical Biology, Indiana University-Purdue University Indianapolis, 402 N. Blackford Street, Indianapolis, IN 46202, USA; modonnel@iupui.edu

**Keywords:** proline and homologues, piperazine derivatives, peptidomimetics, solid-phase organic synthesis, combinatorial chemistry, Distributed Drug Discovery (D3), CNS agent.

## Abstract

A solid-phase procedure is used to synthesize racemic peptidomimetics based on the fundamental peptide unit. The peptidomimetics are constructed around proline or proline homologues variably substituted at the amine and carbonyl sites. The procedure expands the diversity of substituted peptidomimetic molecules available to the Distributed Drug Discovery (D3) project. Using a BAL-based solid-phase synthetic sequence the proline or proline homologue subunit is both constructed and incorporated into the peptidomimetic by an α-alkylation, hydrolysis and intramolecular cyclization sequence. Further transformations on solid-phase provide access to a variety of piperazine derivatives representing a class of molecules known to exhibit central nervous system activity. The procedure works well with proline cores, but with larger six- and seven-membered ring homologues the nature of the carboxylic acid acylating the cyclic amine can lead to side reactions and result in poor overall yields.

## 1. Introduction

**Figure 1:**
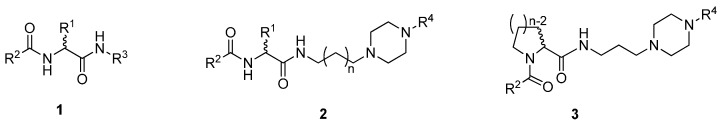
Fundamental peptide unit molecules **1**, **2**, and proline and proline homologues **3**.

A major initiative in the Distributed Drug Discovery (D3) project is the development of simple, inexpensive and reproducible synthetic procedures for access to a large number of biomimetic molecules [1,2,3,4]. Flexible synthetic routes are sought that increase the diversity of molecule types available to D3. We recently reported a solid-phase synthetic route to derivatives of the generic fundamental peptide unit **1** [5]. Many variations of R^1^, R^2^, and R^3^ are permitted on this inherently biomimetic scaffold. We also published a synthetic route to **2** giving access to selective binding agents for subclasses of 5-hydroxytryptamine receptors [5,6,7]. Derivatives of scaffold **2** showed central nervous system activity [6]. We sought a solid-phase route to analogs **3**, of **1** and **2**, where R^1^ was incorporated into a cyclic amine (Figure 1).

**Scheme 1:**
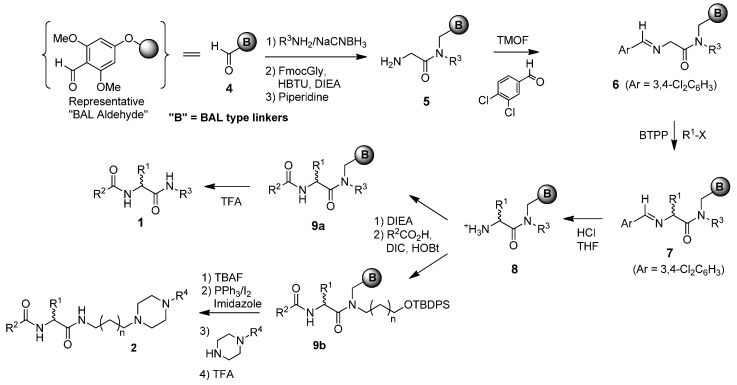
Solid-phase synthetic routes to compounds **1** and **2** using BAL-based resins.

Compounds **1** and **2** can be readily prepared on solid support using BAL-type resins [5,7]. The reactions of this 9- and 12-step synthesis are outlined in Scheme 1.

**Scheme 2:**
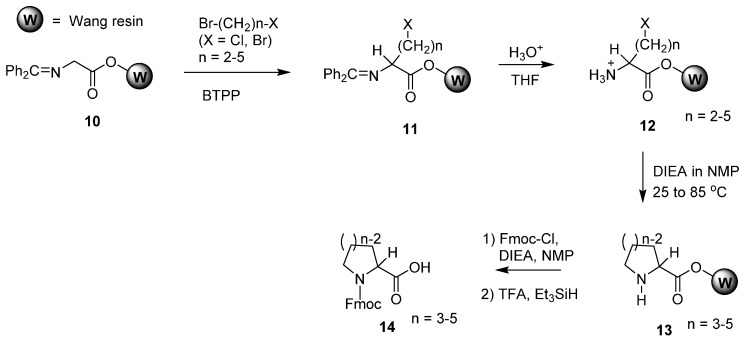
Solid-phase synthesis of proline and homologues from resin-bound intermediate **10** [8,9].

Proline, with its cyclic and secondary amine structure, confers unique properties on proline-containing peptides, proteins, and drugs [8,9]. This is a consequence of conformational constraints induced by this cyclic amino acid residue. It is typically incorporated into peptides by direct acylation of proline in a solid-phase synthetic sequence. To explore the consequences of subtle changes in proline structure on biological activity, we developed a five-step solid-phase synthetic route to racemic proline and proline homologues involving on-resin alkylation with α,ω-dihaloalkanes, followed by hydrolysis and intramolecular cyclization (Scheme 2) [8,9].

This paper describes adaptation of this chemistry to the BAL-based sequence outlined in Scheme 1, permitting the BAL-based solid-phase construction of racemic proline and homologues (compounds **3** in Figure 1) with simultaneous incorporation into the basic peptide unit **1**. An exemplification incorporates piperazine derivatives into **3** to access the unique structural feature of compounds **2** identified as CNS agents. These results provide the research foundation for a highly flexible synthetic route for utilization in future D3-compatible processes.

## 2. Results and Discussion

**Scheme 3:**
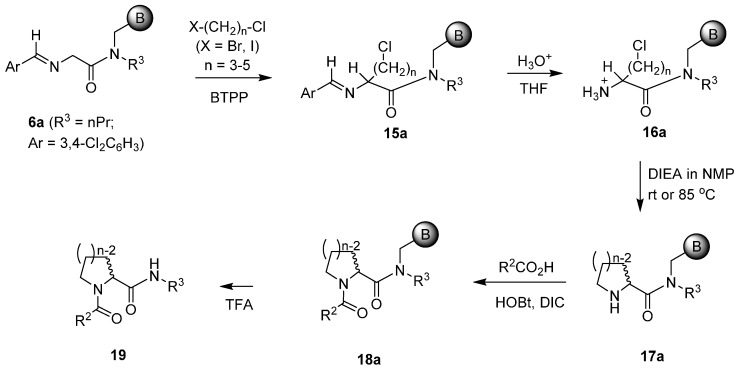
Pilot solid-phase synthetic pathway using BAL-based aldimine resins to yield proline amide and homologues.

Formation of the five-, six- and seven-membered nitrogen-containing rings in this solid-phase sequence relies on successful alkylation of the Schiff base of a resin-bound glycinate on BAL with α,ω-dihaoalkanes and subsequent intramolecular halide displacement by the α-amino group (Scheme 3). Previous studies demonstrated that use of the glycine aldimine provided the required activation for clean monoalkylation without further undesired dialkylation [7]. Therefore, aldimine **6** was chosen as the substrate for alkylation.

Pilot alkylation of aldimine **6a** (R^3^ = nPr) with 1-bromo-3-chloropropane, hydrolysis to **16a**, cyclization to **17a**, followed by acylation with 4-cyanobenzoic acid and cleavage gave **19** (R^3^ = nPr; R^2^ = 4-NC-C_6_H_4_) along with side products with an α-allyl group. This suggested a competing alkylation with allyl halide, an activated electrophile, resulted from *in situ* elimination of 1-bromo-3-chloropropane. This undesired elimination and subsequent alkylation reaction was alleviated when 1-chloro-3-iodo-propane was used and completely absent when propane dihalides were replaced with butane or pentane dihalides, indicating expected nucleophilic displacement was dominating when allyl halide species were not produced (Table 1). Products for this nine-step synthesis (from BAL-resin **4**) were obtained in moderate to good overall yields (Table 1). A higher yield to the bicyclic compound **19d** was obtained when an activated electrophile α,α′-dichloro-*p*-xylene was used as the alkyl dihalide (R^1^X).

**Scheme 4:**
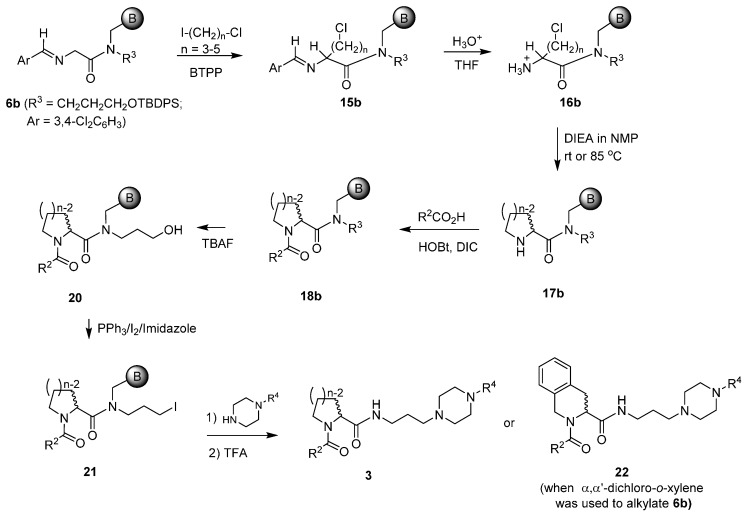
Solid-phase synthetic route (using BAL-based aldimine resins) to proline and homologues substituted on both the amine and carbonyl residues.

Encouraged by these initial results for this nine-step synthetic pathway to cyclic amino acid amide derivatives, we next explored the chemical compatibility of on-resin cyclized intermediate products with: (1) acylations using more hindered carboxylic acids (cyclopropyl carboxylic acid and 1-admantane carboxylic acid), and (2) the subsequent on-resin nucleophilic displacement reaction of iodo-containing intermediates **21** with piperazines (Scheme 4). This nucleophilic displacement sequence was performed with the previously developed method of alcohol activation using PPh_3_/I_2_/imidazole [5]. Studies to target compounds **3** and **22** were conducted with the same four alkylating agents used for the preparation of compounds **19**; cyclopropyl carboxylic acid and 1-admantane carboxylic acid in place of 4-cyanobenzoic acid; and three substituted piperazines. For *n* = 3, moderate overall yields of **3** and **22** were obtained in this 12-step synthesis (based on starting material **4**) (Table 2).

**Scheme 5:**
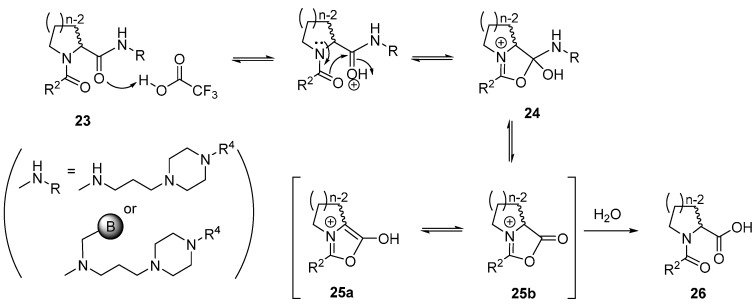
Proposed mechanism for the hydrolysis of proline/proline homologue intermediate products upon treatment with trifluoroacetic acid (TFA).

In contrast to the successful preparation of six- and seven-membered aliphatic ring homologs in the pilot Scheme 3, these scaffolds were not obtained (Scheme 4) when either cyclopropyl carboxylic acid or 1-admantane carboxylic acid was used in place of 4-cyanobenzoic acid. However, when α,α′-dichloro-*p*-xylene was used as the alkylating agent the desired final products were obtained in moderate yields with cyclopropane carboxylic acid as the acylating agent and piperazines of higher nucleophilicity. Based on the initial results shown in Table 1, five-, six-, and seven-membered rings should also have been formed by the sequence of alkylation, hydrolysis and intramolecular cyclization (Scheme 4). Therefore, the absence of the desired six- and seven-membered ring products can be attributed to reactions other than alkylation. This is likely due to an unstable amide bond to the α-carboxylic acid, a function, in these compounds, of *both* the ring size and the acylating agent. Unexpected hydrolysis of the amide bond involving the carboxylate of the six-membered ring pipecolic acid residue has been reported during TFA treatment of peptides [10]. Hydrolytic instability with electron donating acylating agents has also been reported [11]. In both these reports hydrolysis was proposed to proceed via an oxazolinium ion intermediate mechanism (Scheme 5). The ease of the amide bond hydrolysis is highly dependent on the stability of the intermediate **25a**/**25b**, which is affected by two factors: the size of the ring that is fused to the oxazolinium ion (steric effect) [10] and the electronic nature of the R^2^ group (electronic effect) [11]. Compared with the five-membered ring, six- or seven-membered rings are less constrained, thus favoring the formation of the oxazolinium ion leading to the hydrolysis. In addition, electron-donating substituents on the acyl residue R^2^ stabilize the oxazolinium cation, thus favoring its formation and promoting hydrolysis. The combination of these steric and electronic effects magnifies the hydrolytic instability. These results are consistent with the successful preparation of the five, six and seven-membered compounds **19** (Table 1) where, in spite of the larger ring sizes, the electron-withdrawing cyano group on the aromatic acylating group destabilizes the oxazolinium cation, and, therefore, disfavors the hydrolytic side reaction to afford expected products (**19**).

## 3. Experimental Section

### 3.1. General

Solution and solid-phase organic transformations and resin washes were carried out at ambient temperature, unless indicated otherwise. Organic solvents and reagents were of reagent grade and used directly without purification. Chloroform-*d*_1_, 1-chloro-3-iodopropane, 1-(3-chlorophenyl)piperazine, α,α′-dichloro-*o*-xylene, 1,3-diisopropylcarbodiimide (DIC), anhydrous *N*,*N*-dimethylforamide (DMF), and sodium bisulfate monohydrate (NaHSO_4_·H_2_O) were purchased from Acros Organics. Acetic acid, trifluoroacetic acid (TFA), hydrochloric acid, and methanol were obtained from Fisher Scientific (Waltham, MA, USA). 1-Adamantane carboxylic acid was purchased from Fluka. 3-Aminopropanol, *tert*-butyl(chloro)diphenylsilane, 1-chloro-4-iodobutane, 1-chloro-5-iodopentane, 4-cyanobenzoic acid, 3,4-dichlorobenzaldehyde, 1-(2,3-dichlorophenyl)-piperazine hydrochloride, *N,N*-diisopropylethylamine (DIEA), 1-hydroxybenzotriazole hydrate (HOBt), imidazole, methanol-*d*_4_, 1-(2-methoxyphenyl)piperazine, piperidine, sodium cyanoborohydride, tetrabutylammonium fluoride (TBAF) (1M solution in THF), triethylamine, triphenylphosphine and trimethylorthoformate were obtained from Aldrich Chemical Co. (St. Louis, MO, USA) 4-Methylbenzhydrylamine hydrochloride resin (PL-MBHA·HCl, 1.6 mmol/g, 75–150 μm) was purchased from Polymer Laboratories (Hopkins, MN, USA). 4-(4-Formyl-3,5-dimethoxyphenoxy)butyric acid (BAL linker), *N*-α-Fmoc-glycine and 2-(1*H*-Benzotriazole-1-yl)-1,1,3,3-tetramethylaminium hexafluorophosphate (HBTU) were purchased from NovaBiochem (Danvers, MA, USA). Iodine was obtained from J. T. Baker (Avantor Performance Materials, Center Valley, PA, USA) and used without further purification. *O*-*t*-Butyldiphenylsilyl-3-aminopropanol hydrochloride (TBDPSO(CH_2_)_3_NH_2_·HCl) was prepared following the reported procedure [12]. Triphenylphosphine was recrystallized from hexanes before use. Ratios in all solvent and reagent mixtures prepared are volume to volume unless otherwise noted.

Manual solid-phase organic syntheses were carried out in two types of reaction vessels. For large scale (up to 3.7 mmol) reactions peptide synthesis reaction vessels (50 mL) with coarse porosity fritted glass support and supplied with a GL thread and a Teflon-lined PBT screw cap (Chemglass, CG-1860-03, Chemglass Life Sciences, Vineland, NJ, USA) were used. For small-scale reactions (typically 50 µmol each) Bill-Board equipment (Chemglass Life Sciences), designed to be inexpensive, simplify and expedite multiple, manual solid-phase syntheses [2,4,13], was used. The complete Bill-Board set (Catalog number CG-1869) and associated replacement parts and supplies can be purchased from Chemglass. For agitation, the large-scale reactions in the peptide synthesizers were placed on an orbital shaker (Thermolyne, Roto Mix, Type 50800, Alpha Multiservices Inc. Conroe, TX, USA) while appropriate motor rotators were used as rotation devices for small-scale reactions. 

Depending on the number of reactions to be conducted, the starting resin was distributed either by weight or as aliquots from an isopycnic suspension [2]. In the case of distribution by volume from an isopycnic suspension, the Bill-Boards were placed in their drain trays, and from a neutral buoyancy suspension in CH_2_Cl_2_-NMP typically, 50 μmols of the starting resin (with a known loading) was distributed, via repeated aliquots (1 mL), to each of the reaction vessels in a given Bill-Board (6-pack or 24-pack). During the distribution of the resin, the isopycnic solvent was allowed to drain through the frit in the reaction vessels. When distribution was complete, residual solvent was removed with an “air-push” from a disposable plastic pipet (Fisher, 13-711-23) fitted with a pierced septum (Aldrich, Z12743-4). The resin was then washed with an appropriate solvent (this solvent wash was also carried out when the resin was weighed into the reaction vessels). The bottom of each reaction vessel was then capped, and a new calibrated pipet (Fisher, 13-711-24) was used for adding each reagent in the following step. The tops of all reaction vessels were capped and the Bill-Board was placed on an appropriate rotation apparatus. Following the reaction the reagents and solvents were drained and the resin product was then washed with the indicated solvents. Resin-bound intermediates were air-dried after the final CH_2_Cl_2_ washes, unless re-weighing was necessary, in which case overnight drying was carried out under high vacuum (≤0.2 mmHg) or under low vacuum (house vacuum) for 24–36 h in a vacuum desiccator. During resin washing with solvents for large scale reactions, at least 3 min of solvent contact with the resin in the reaction vessels (bottom closed) was performed, then the resin was drained, followed by an air-push. For washing of small scale reactions, at least 30 s was normally used after addition of solvents to the reaction vessels (with bottom end open for draining) followed by an air-push. Solid-phase reactions at elevated temperatures (50 °C, 60 °C and/or 80 °C) were carried out in an Isotemp^®^ Oven Model 280A (Fisher Scientific) with the reaction vessels capped to finger tightness.

Analytical thin layer chromatography (TLC) was performed with EM Science silica gel 60 F_254_, 0.25 mm pre-coated glass plates (EMD Chemical Inc., 5715-7, Gibbstown, NJ, USA). TLC plates were visualized using UV_254_. Column chromatography was performed on HyperSep SI^®^ 3 mL cartridges (60108-315, Thermo Fisher Scientific Inc.) preloaded with 500 mg of silica gel 60 (irregular particles 40–63 μm) from Thermo Electron Corporation (Beverly, MA, USA). The yields of the final compounds, after chromatographic purification, were calculated on the basis of the initial loading of the starting resins and are the overall yields for all reaction steps starting from these resins. ^1^H-NMR (500 MHz) and ^13^C-NMR (125 MHz) spectra were recorded on a Bruker Avance III 500 spectrometer (Billerica, MA, USA). Chemical shifts are reported in parts per million (ppm) and are referenced to the centerline of chloroform-*d*_1_ (δ 7.26 ppm ^1^H-NMR, 77.0 ppm ^13^C-NMR) using TMS (0.00 ppm), or chloroform-*d*_1_ mixed with methanol-*d*_4_ (2%–10%). Coupling constants are given in Hertz (Hz).

Electrospray ionization mass spectrometry was conducted using a PESciex API III triple stage quadrupole mass spectrometer (Agilent Technologies, Santa Clara, CA, USA) operated in either positive-ion or negative-ion detection mode. LC/MS analyses for initial purity of the crude products were conducted using an Agilent system (Agilent Technologies), consisting of a 1100 series HPLC connected to a diode array detector and a 1946D mass spectrometer configured for positive-ion/negative-ion electrospray ionization. The LC/MS samples were analyzed as solutions in CH_3_CN, prepared at 0.08–0.12 mg/mL concentration. The LC/MS derived composition of mixtures was determined based on UV integration at 210 nm. The LC/MS chromatography was carried out on an Agilent Zorbax SB-C_8_ column (PN835975-906; 4.6 mm × 50 mm, 3.5 μm) with linear gradients of 0.1% TFA in CH_3_CN and 0.1% aqueous TFA and were run at 1.0 mL/min flow rate from 20:80 to 90:10 for 25 min. The composition of reaction mixtures was determined based on the integration of NMR spectra as well as LC/MS results. High-resolution mass spectrometry was obtained on a MAT 95XP (Thermo Electron Corp.) with chemical ionization (CI), electron impact (EI), or fast-atom bombardment (FAB) mode.

### 3.2. General Procedures for Manual Solid-Phase Organic Syntheses

#### 3.2.1. Preparation of MBHA-BAL Resin **4** and Derivatives **6a** and **6b**

The preparations followed the reported procedures [5,7].

#### 3.2.2. General Procedures for the Preparation of **17a** and **17b** from **6a** and **6b**

Alkylations of the Aldimine of Glycine on BAL Resins **6a** or **6b** with α,ω-Dihaloalkanes, Subsequent Imine Hydrolysis, and Intramolecular Cyclization: After the resin bound Schiff base **6a** or **6b** (50 μmol) was washed with CH_2_Cl_2_ (3 × 1 mL) and pre-swelled in CH_2_Cl_2_ (1 mL) for 1 h, it was washed with NMP (4 × 1 mL). To the resin was added BTPP solutions in NMP (1.0 M, 0.5 mL, 10 eq), followed by addition of I-(CH_2_)_n_-Cl (neat, 0.5 mmol, 10 eq, whereas in the case of α,α’-dichloro-*o*-xylene, its solution in NMP (0.25 mL, 2.0 M, 10 eq) was added). Alkylation was allowed to proceed at room temperature for 24 h with rotation. The alkylated resin product **15a** or **15b** was washed with NMP (4 × 1 mL), CH_2_Cl_2_ (4 × 1 mL), and THF (4 × 1 mL), the resin was then hydrolyzed to afford **16a** or **16b** using 1N HCl-THF (1:2, 1 mL) over 20 min. After the drained resin was washed with THF (3 × 1 mL), CH_2_Cl_2_ (3 × 1 mL), and NMP (3 × 1 mL), it was then treated with 10% DIEA/NMP (1 mL) at ambient temperature for 24 h. The resulting resin bound product **17a** or **17b** was washed with NMP (4 × 1 mL), THF (3 × 1 mL), and CH_2_Cl_2_ (4 × 1 mL).

#### 3.2.3. General Procedures for the Preparation of **19** from **17a**

After the resin **17a** was swelled in CH_2_Cl_2_ for 1 h and washed with DMF (4 × 1 mL), it was treated with a pre-mixed clear solution of carboxylic acid (0.25 mmol, 5 eq), HOBt (0.034 g, 0.25 mmol, 5 eq) and DIC (0.032 g, 0.25 mmol, 5 eq) in DMF. The completion of the reaction was indicated by a negative chloranil test [14]. The resulting resin product **18a** was then washed with DMF (4 × 1 mL), THF (3 × 1 mL), and CH_2_Cl_2_ (3 × 1 mL). Cleavage with 90% TFA/CH_2_Cl_2_ (1 mL) at room temperature over 1.5 h gave desired product **19a**–**d**. (Scheme 3).

*1-(4-Cyanobenzoyl)-N-propylpyrrolidine-2-carboxamide* (**19a**): Product **19a** was obtained from resin **6a**, 1-chloro-3-iodopropane, and 4-cyanobenzoic acid as light yellow oil (4.0 mg, 28% isolated yield) following chromatographic purification over silica gel with CH_2_Cl_2_/MeOH (97:3). Initial LC/MS purity (for crude product) 43%, *t*_R_ = 3.1 min. ^1^H-NMR (500 MHz, CDCl_3_): δ 0.93 (t, *J* = 7.3 Hz, 3H), 1.53 (tq, *J* = 7.3 Hz, *J* = 7.3 Hz, 2H), 1.82–1.89 (m, 1H), 1.96–2.09 (m, 3H), 2.43–2.52 (m, 1H), 3.20–3.28 (m, 2H), 3.34–3.44 (m, 1H), 3.49–3.58 (m, 1H), 4.68 (dd, *J* = 7.3 Hz, *J* = 3.2 Hz, 1H), 6.78 (brs, 1H), 7.62 (d, *J* = 8.5 Hz, 2H), 7.72 (d, *J* = 8.6 Hz, 2H). ^13^C-NMR (500 MHz, CDCl_3_): δ 11.4, 20.4, 22.7, 25.8, 41.4, 46.0, 52.7, 113.9, 117.9, 126.5, 132.5, 139.7, 170.3, 171.1.

*1-(4-Cyanobenzoyl)-N-propylpiperidine-2-carboxamide* (**19b**): Product **19b** was obtained from resin **6a**, 1-chloro-4-iodobutane, and 4-cyanobenzoic acid as light yellow oil (3.0 mg, 20% isolated yield) following chromatographic purification over silica gel with CH_2_Cl_2_/MeOH (98:2). Initial LC/MS purity (for crude product) 46%, *t*_R_ = 5.9 min. ^1^H-NMR (500 MHz, CDCl_3_): δ 0.92 (t, *J* = 7.3 Hz, 3H), 1.46–1.58 (m, 4H), 1.60–1.76 (m, 2H), 1.83–1.93 (m, 1H), 2.22–2.32 (m, 1H), 3.15–3.35 (m, 3H), 3.46–3.55 (m, 1H), 5.20 (m, 1H), 6.32 (brs, 1H), 7.53 (d, *J* = 8.5 Hz, 2H), 7.72 (d, *J* = 8.5 Hz, 2H). ^13^C-NMR (500 MHz, CDCl_3_): δ 11.2, 21.0, 23.1, 23.8, 25.2, 42.5, 44.5, 62.3, 113.6, 118.1, 127.9, 132.1, 139.5, 170.5, 171.3.

*1-(4-Cyanobenzoyl)-N-propylazepane-2-carboxamide* (**19c**): Product **19c** was obtained from resin **6a**, 1-chloro-5-iodopentane, and 4-cyanobenzoic acid as light yellow oil (4.0 mg, 26% isolated yield) following chromatographic purification over silica gel with CH_2_Cl_2_/MeOH (98:2). Initial LC/MS purity (for crude product) 82%, *t*_R_ = 6.1 min. ^1^H-NMR (500 MHz, CDCl_3_): δ 0.93 (t, *J* = 7.3 Hz, 3H), 1.34–1.40 (m, 4H), 1.49–1.60 (m, 2H), 1.97–2.21 (m, 4H), 3.18–3.29 (m, 2H), 3.37–3.45 (m, 2H), 4.85 (dd, *J* = 7.4 Hz, *J* = 3.0 Hz, 1H), 6.32 (brs, 1H), 7.47 (d, *J* = 8.5 Hz, 2H), 7.71 (d, *J* = 8.6 Hz, 2H). ^13^C-NMR (500 MHz, CDCl_3_): δ 11.5, 23.0, 25.2, 28.9, 29.3, 30.9, 41.4, 46.2, 58.5, 113.5, 118.3, 127.1, 132.7, 141.2, 171.5, 172.2.

*2-(4-Cyanobenzoyl)-N-propyl-1,2,3,4-tetrahydroisoquinoline-3-carboxamide* (**19d**): Product **19d** was obtained from resin **6a**, α,α′-dichloro-*o*-xylene, and 4-cyanobenzoic acid as light yellow oil (9.0 mg, 52% isolated yield) following chromatographic purification over silica gel with CH_2_Cl_2_/MeOH (99:1). Initial LC/MS purity (for crude product) 75%, *t*_R_ = 7.9 min. ^1^H-NMR (500 MHz, CDCl_3_): δ 0.91 (t, *J* = 7.3 Hz, 3H), 1.52 (m, 2H), 3.10–3.18 (m, 1H), 3.19–3.32 (m, 2H), 3.42–3.49 (m, 1H), 4.33–4.54 (m, 2H), 5.13 (m, 1H), 6.46 (brs, 1H), 7.16–7.28 (m, 4H), 7.54 (d, *J* = 8.5 Hz, 2H), 7.77 (d, *J* = 8.5 Hz, 2H). ^13^C-NMR (500 MHz, CDCl_3_): δ 11.3, 22.8, 29.7, 41.4, 49.6, 53.9, 114.1, 117.9, 125.2, 126.8, 127.5, 127.8, 128.2, 128.5, 133.2, 133.9, 139.7, 169.9, 170.1.

#### 3.2.4. General Procedures for the Preparation of **3** and **22** from **17b**

Synthesis of New Proline Amide and Homologue -based Piperazine Incorporated Products **3** and **22** through Acylation, Deprotection, Iodination, *N*-Alkylation with Piperazines, and Cleavage: After the resin **17b** was swelled in CH_2_Cl_2_ for 1 h and washed with DMF (4 × 1 mL), it was treated with a pre-mixed clear solution of carboxylic acid (0.25 mmol, 5 eq), HOBt (0.034 g, 0.25 mmol, 5 eq) and DIC (0.032 g, 0.25 mmol, 5 eq) in DMF. The completion of the reaction was indicated by a negative chloranil test [14]. The resulting resin product **18b** was then washed with DMF (4 × 1 mL), THF (3 × 1 mL), and CH_2_Cl_2_ (3 × 1 mL). After **18b** was pre-swelled in CH_2_Cl_2_ for 40 min, it was washed with THF (3 × 1 mL), followed by treatment with 1M TBAF/THF (1 mL, 1 mmol, 20 eq) for removal of TBDPS group. After the reaction proceeded for 18 h, the drained resin product **20** was washed with THF (4 × 1 mL), and CH_2_Cl_2_ (3 × 1 mL). Iodination of alcohol resin **20** started with its pre-swelling in CH_2_Cl_2_ for 1 h, followed by washes with DMF (3 × 1 mL). The resin was then treated with a pre-mixed solution of iodine (0.063 g, 0.25 mmol, 5 eq), PPh_3_ (0.066 g, 0.25 mmol, 5 eq), and imidazole (0.017 g, 0.25 mmol, 5 eq) in DMF (0.75 mL). The reaction was allowed to proceed at room temperature overnight. After 18 h of iodination reaction, the filtered iodinated resin product **21** was washed with DMF (3 × 1 mL), MeOH (3 × 1 mL), DMF (2 × 1 mL), and CH_2_Cl_2_ (3 × 1 mL). The air-dried resin was swollen in CH_2_Cl_2_ for 40 min, and to the resin was then added a solution of Piperazines in DMSO (1 M, 0.75 mL, 15 eq) except that 2,3-dichlorophenyl piperazine hydrochloride (0.201 g, 0.75 mmol, 15 eq) was added as the solid together with same equivalent of base DIEA (0.097 g, 0.75 mmol, 15 eq). The reaction was heated to 50 °C for 6 h with occasional agitation. The resulting resin product was drained, and sequentially washed with DMF (2 × 1 mL), MeOH (2 × 1 mL), DMF (3 × 1 mL), and CH_2_Cl_2_ (4 × 1 mL). Cleavage with 90% TFA/CH_2_Cl_2_ (1 mL) at room temperature over 1.5 h afforded target compound **3a**–**3d**, **22a** and **22b**.

*1-(Cyclopropanecarbonyl)-N-(3-(4-(2-methoxyphenyl)piperazin-1-yl)propyl)pyrrolidine-2-carboxamide* (**3a**): Product **3a** was obtained from resin **6b**, 1-chloro-3-iodopropane, cyclopropanecarboxylic acid, and 1-(2-methoxyphenyl)piperazine as light yellow oil (2.9 mg, 14% isolated yield) following chromatographic purification over silica gel with CH_2_Cl_2_/MeOH (95:5). Initial LC/MS purity (for crude product) 49%, *t*_R_ = 3.3 min. ^1^H-NMR (500 MHz, CDCl_3_): δ 0.78–0.82 (m, 2H), 0.83–0.89 (m, 1H), 0.91–0.99 (m, 1H), 1.69–1.79 (m, 1H), 1.98–2.22 (m, 8H), 2.98–3.05 (m, 2H), 3.24–3.35 (m, 4H), 3.43–3.52 (m, 3H), 3.64–3.69 (m, 2H), 3.87 (s, 3H), 3.89–3.94 (m, 1H), 4.44 (dd, *J* = 7.6 Hz, *J* = 3.0 Hz, 1H), 6.89 (d, *J* = 7.9 Hz, 1H), 6.93–6.96 (m, 2H), 7.04–7.11 (m, 1H), 7.83 (m, 1H). ^13^C-NMR (500 MHz, CDCl_3_): δ 7.5, 8.0, 12.8, 23.0, 24.8, 28.9, 35.8, 47.3, 47.6, 51.8, 53.1, 54.2, 55.5, 60.9, 111.4, 118.9, 121.3, 124.5, 139.0, 152.2, 162.1, 173.0, 173.9.

*N-(3-(4-(3-Chlorophenyl)piperazin-1-yl)propyl)-1-(cyclopropanecarbonyl)pyrrolidine-2-carboxamide* (**3b**): Product **3b** was obtained from resin **6b**, 1-chloro-3-iodopropane, cyclopropanecarboxylic acid, and 1-(3-chlorophenyl)piperazine as light yellow oil (3.0 mg, 14% isolated yield) following chromatographic purification over silica gel with CH_2_Cl_2_/MeOH (92:8). Initial LC/MS purity (for crude product) 43%, *t*_R_ = 5.1 min. ^1^H-NMR (500 MHz, CDCl_3_): δ 0.79–0.89 (m, 3H), 0.90–0.97 (m, 1H), 1.69–1.78 (m, 1H), 1.94–2.26 (m, 8H), 2.84–2.95 (m, 1H), 2.98–3.07 (m, 1H), 3.22–3.33 (m, 2 H), 3.38–3.49 (m, 3 H), 3.57–3.72 (m, 4H), 3.86–3.93 (m, 1H), 4.42 (dd, *J* = 7.5 Hz, *J* = 3.1 Hz), 6.79 (dd, *J* = 8.3 Hz, *J* = 2.2 Hz, 1H), 6.89 (m, 1H), 6.92–6.97 (m, 1H), 7.21 (t, *J* = 8.1 Hz, 1H), 7.73 (t, *J* = 5.2 Hz). ^13^C-NMR (500 MHz, CDCl_3_): δ 7.5, 8.1, 12.8, 23.1, 24.8, 29.0, 35.9, 46.3, 47.6, 54.3, 60.9, 114.9, 117.2, 121.5, 130.5, 135.3, 150.6, 173.0, 173.9. HRMS calcd for [M + H]^+^: C_22_H_32_N_4_O_2_Cl 419.2214, found 419.2229.

*1-(Cyclopropanecarbonyl)-N-(3-(4-(2,3-dichlorophenyl)piperazin-1-yl)propyl)pyrrolidine-2-carboxamide* (**3c**): Product **3c** was obtained from resin **6b**, 1-chloro-3-iodopropane, cyclopropanecarboxylic acid, and 1-(2,3-dichlorophenyl)-piperazine hydrochloride as light yellow oil (2.0 mg, 9% isolated yield) following chromatographic purification over silica gel with CH_2_Cl_2_/OH (95:5). Initial LC/MS purity (for crude product) 41%, *t*_R_ = 6.1 min. ^1^H-NMR (500 MHz, CDCl_3_): δ 0.79–0.89 (m, 3H), 0.91–0.97 (m, 1H), 1.69–1.78 (m, 1H), 1.99–2.05 (m, 2H), 2.06–2.11 (m, 2H), 2.14–2.22 (m, 2H), 2.97–3.09 (m, 3H), 3.26–3.34 (m, 2H), 3.36–3.46 (m, 5H), 3.63–3.74 (m, 3H), 3.87–3.94 (m, 1H), 4.43 (dd, *J* = 7.6 Hz, *J* = 3.1 Hz, 1H), 7.02 (dd, *J* = 8.0 Hz, *J* = 1.4 Hz, 1H), 7.19 (t, *J* = 8.0 Hz, 1H), 7.25 (d, *J* = 1.3 Hz, 1H), 7.75 (t, *J* = 5.6 Hz, 1H). ^13^C-NMR (500 MHz, CDCl_3_): 7.5, 8.1, 12.8, 23.1, 24.8, 29.0, 35.8, 47.6, 47.9, 54.4, 60.9, 119.2, 126.2, 127.8, 127.9, 134.3, 149.1, 162.4, 173.0, 173.9. HRMS calcd for [M + H]^+^: C_22_H_31_N_4_O_2_Cl_2_ 453.1824, found 453.1845 (RA 100%).

*1-(Adamantanecarbonyl)-N-(3-(4-(2-methoxyphenyl)piperazin-1-yl)propyl)pyrrolidine-2-carboxamide* (**3d**): This product was obtained from resin **6b**, 1-chloro-3-iodopropane, 1-adamantanecarboxylic acid, and 1-(2-methoxyphenyl)piperazine as light yellow oil (5.7 mg, 22% isolated yield) following chromatographic purification over silica gel with CH_2_Cl_2_/MeOH (92:8). Initial LC/MS purity (for crude product) 44%, *t*_R_ = 9.3 min. ^1^H-NMR (500 MHz, CDCl_3_): δ 1.71 (m, 6H), 1.85–1.95 (m, 3H), 1.96–1.99 (m, 5H), 2.00–2.09 (m, 7H), 3.05 (t, *J* = 10.8 Hz, 2H), 3.13–3.18 (m, 1H), 3.20–3.32 (m, 4H), 3.41–3.47 (m, 1H), 3.48–3.54 (m, 2H), 3.66 (d, *J* = 11.8 Hz, 1H), 3.76–3.84 (m, 3H), 3.87 (s, 3H), 4.43 (dd, *J* = 7.4 Hz, *J* = 5.3 Hz, 1H), 6.89 (d, *J* = 8.1 Hz, 1H), 6.92–6.95 (m, 2H), 7.03–7.12 (m, 1H), 7.41 (m, 1H). ^13^C-NMR (500 MHz, CDCl_3_): δ 23.7, 28.3, 35.9, 36.6, 38.2, 41.8, 47.5, 48.6, 52.4, 52.6, 54.6, 55.5, 62.9, 111.4, 118.9, 121.2, 124.4, 139.0, 152.2, 162.5, 173.9, 176.9. HRMS calcd for [M + H]^+^: C_30_H_45_N_4_O_3_ 509.3492, found 509.3474.

*N-(3-(4-(3-Chlorophenyl)piperazin-1-yl)propyl)-1-(adamantanecarbonyl)pyrrolidine-2-carboxamide* (**3e**): This product was obtained from resin **6b**, 1-chloro-3-iodopropane, 1-adamantanecarboxylic acid, and 1-(3-chlorophenyl)piperazine as light yellow oil (2.0 mg, 8% isolated yield) following chromatographic purification over silica gel with CH_2_Cl_2_/MeOH (92:8). Initial LC/MS purity (for crude product) 39%, *t*_R_ = 10.8 min. ^1^H-NMR (500 MHz, CDCl_3_): δ 1.63–1.78 (m, 6H), 1.89–1.98 (m, 7H), 1.99–2.12 (m, 8H), 2.95 (m, 2H), 3.12–3.20 (m, 1H), 3.21–3.31 (m, 2H), 3.36–3.52 (m, 3H), 3.56–3.71 (m, 3H), 3.78–3.89 (m, 3H), 4.38 (dd, *J* = 7.7 Hz, *J* = 5.2 Hz, 1H), 6.78 (dd, *J* = 8.2 Hz, *J* = 2.1 Hz, 1H), 6.86–6.90 (m, 1H), 6.91–6.95 (m, 1H), 7.16–7.24 (m, 2H). ^13^C-NMR (500 MHz, CDCl_3_): δ 23.8, 28.3, 36.1, 36.6, 38.2, 41.8, 46.5, 48.6, 54.5, 62.9, 114.9, 117.2, 121.4, 130.4, 135.3, 150.6, 173.9, 176.9. HRMS calcd for [M + H]^+^: C_29_H_42_N_4_O_2_Cl 513.2996, found 513. 2987.

*1-(Adamantanecarbonyl)-N-(3-(4-(2,3-dichlorophenyl)piperazin-1-yl)propyl)pyrrolidine-2-carboxamide* (**3f**): This product was obtained from resin **6b**, 1-chloro-3-iodopropane, 1-adamantanecarboxylic acid, and 1-(2,3-dichlorophenyl)-piperazine hydrochloride as light yellow oil 2.0 mg (7% isolated yield) following chromatographic purification (CH_2_Cl_2_/MeOH (92:8)): initial LC/MS purity (for crude product) 39%, *t*_R_ = 11.5 min. ^1^H-NMR (500 MHz, CDCl_3_): δ 1.66–1.77 (m, 6H), 1.91–1.99 (m, 7H), 2.00–2.13 (m, 8H), 3.00–3.12 (m, 2H), 3.14–3.23 (m, 1H), 3.23–3.31 (m, 2H), 3.35–3.42 (m, 4H), 3.45–3.55 (m, 1H), 3.65–3.73 (m, 1H), 3.78–3.89 (m, 3H), 4.39 (dd, *J* = 7.7 Hz, *J* = 5.3 Hz, 1H), 7.01 (dd, *J* = 7.9 Hz, *J* = 1.1 Hz, 1H), 7.19 (t, *J* = 7.9 Hz, 2H), 7.24 (m, 1H). ^13^C-NMR (500 MHz, CDCl_3_): δ 23.9, 28.3, 35.9, 36.6, 38.2, 41.8, 48.2, 48.7, 54.7, 62.9, 119.2, 126.2, 127.8, 127.9, 134.3, 149.1, 173.8, 176.9. HRMS calcd for [M + H]^+^: C_29_H_41_N_4_O_2_Cl_2_ 547.2607, found 547.2627.

*2-(Cyclopropanecarbonyl)-N-(3-(4-(2-methoxyphenyl)piperazin-1-yl)propyl)-1,2,3,4-tetrahydroisoquinoline-3-carboxamide* (**22a**): Product **22a** was obtained from resin **6b**, α,α′-dichloro-*o*-xylene, cyclopropanecarboxylic acid, and 1-(2-methoxyphenyl)piperazine as amorphous white solid (6.0 mg, 25% isolated yield) following chromatographic purification over silica gel with CH_2_Cl_2_/MeOH (92:8). Initial LC/MS purity (for crude product) 65%, *t*_R_ = 6.2 min. ^1^H-NMR (500 MHz, CDCl_3_, 1:1 mixture of two rotamers): δ 0.78–0.91 (m, 2H), 0.93–1.05 (m, 1H), 1.05–1.19 (m, 1H), 1.38–1.49 (m, 1H), 1.56–1.61 (m, 1H), 1.69–1.83 (m, 2H), 1.90–2.05 (m, 1H), 2.07–2.23 (m, 1H), 2.47–2.59 (m, 2H), 2.63–2.75 (m, 2H), 2.97–3.05 (m, 1H), 3.07–3.16 (m, 3H), 3.17–3.26 (m, 2H), 3.35–3.45 (m, 1H), 3.86 (s, 3H), 4.56 (d, *J* = 15.9 Hz, 0.5 H), 4.81 (d, *J* = 15.9 Hz, 1H), 4.89 (m, 0.5H), 4.94 (d, *J* = 15.0 Hz, 0.5H), 5.16 (m, 0.5H), 6.87 (d, *J* = 7.8 Hz, 1H), 6.90–6.96 (m, 2H), 6.96–7.03 (m, 2H), 7.13–7.25 (m, 4H). ^13^C-NMR (500 MHz, CDCl_3_): δ 8.2, 12.4, 23.9, 29.7, 30.9, 32.9, 44.5, 48.5, 50.5, 53.4, 55.4, 111.2, 118.3, 121.0, 125.8, 126.4, 126.6, 127.3, 127.6, 127.9, 128.5, 133.9, 152.3, 173.3, 175.5. HRMS calcd for [M + H]^+^: C_28_H_37_N_4_O_3_ 477.2866, found 477.2879.

*N-(3-(4-(3-Chlorophenyl)piperazin-1-yl)propyl)-2-(cyclopropanecarbonyl)-1,2,3,4-tetrahydroisoquinoline-3-carboxamide* (**22b**): Product **22b** was obtained from resin **6b**, α,α′-dichloro-*o*-xylene, cyclopropanecarboxylic acid, and 1-(3-chlorophenyl)piperazine as amorphous white solid (6.0 mg, 25% isolated yield) following chromatographic purification over silica gel with CH_2_Cl_2_/MeOH (92:8). Initial LC/MS purity (for crude product) 64%, *t*_R_ = 7.8 min. ^1^H-NMR (500 MHz, CD_3_OD/CDCl_3_, 1 : 1 mixture of two rotamers): δ 0.87–0.91 (m, 2H), 1.06–1.12 (m, 1H), 1.39–1.44 (m, 2H), 1.59–1.66 (m, 2H), 1.85–1.93 (m, 1H), 1.97–2.05 (m, 2H), 2.68–2.76 (m, 1H), 3.02–3.09 (m, 1H), 3.22–3.28 (m, 2H), 3.32–3.40 (m, 2H), 3.42–2.59 (m, 4H), 3.63–3.71 (m, 1H), 4.86 (d, *J* = 15.0 Hz, 1H), 4.91–4.94 (m, 0.5H), 4.99–5.05 (m, 1H), 5.05–5.11 (m, 0.5H), 6.78 (dd, *J* = 8.2 Hz, *J* = 2.4 Hz, 1H), 6.86–6.89 (m, 1H), 6.91–6.95 (m, 1H), 7.14–7.25 (m, 5H). ^13^C-NMR (500 MHz, CD_3_OD/CDCl_3_): δ 12.2, 13.5, 19.7, 21.4, 23.9, 29.8, 46.5, 46.7, 51.8, 58.8, 114.9, 116.9, 126.3, 127.1, 127.8, 130.5, 133.4, 133.7, 134.0, 135.3, 151.0, 175.3, 176.4. HRMS calcd for [M + H]^+^: C_27_H_34_N_4_O_2_Cl 481.2370, found 481.2393.

## 4. Conclusions

In summary, a BAL-based synthetic route to acylated proline amides and six- and seven-membered homologues has been successfully developed using MBHA-BAL resin. Subsequent conversion to scaffolds containing piperazine derivatives was achieved with five-membered prolines but, except in two examples, not in the six- and seven-membered classes. This result appears to be a function of both ring size and the nature of the acylating agent. In order to enable the synthesis of new class of molecules within Distributed Drug Discovery (D3) project, optimization of current reaction conditions and alternative routes to the construction of six-membered or seven-membered proline amide and homologue ring products are under investigation.

## Figures and Tables

**Table 1:** Preliminary studies of alkylating agents for the construction of proline (and homologue) rings using BAL-based aldimine resins. 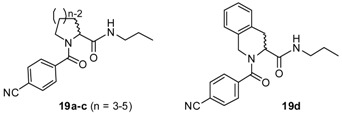

Compound	R^1^X	Yield ^a^
**19a**	I-(CH_2_)_3_-Cl	28%
**19b**	I-(CH_2_)_4_-Cl	20%
**19c**	I-(CH_2_)_5_-Cl	26%
**19d**	α,α′-dichloro-*o*-xylene	52%

^a^ purified overall yields from **4**.

**Table 2:** Survey of eight cyclic amino acid amide compounds successfully prepared from two alkylating agents, two carboxylic acids, and three piperazines.

		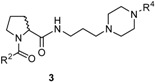		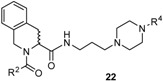
	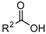	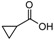	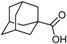	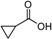
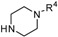				
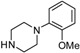		**3a** (14%)	**3d** (22%)	**22a** (25%)
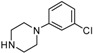		**3b** (14%)	**3e** (8%)	**22b** (25%)
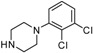		**3c** (9%)	**3f** (7%)	
